# Surveillance and care for confirmed and suspected patients with COVID-19 in general practice (CovidCare): study protocol for an observational trial

**DOI:** 10.1186/s12875-021-01515-8

**Published:** 2021-09-02

**Authors:** Mariell Hoffmann, Sandra Stengel, Johanna Forstner, Annika Baldauf, Gunter Laux, Frank Aluttis, Markus Qreini, Peter Engeser, Joachim Szecsenyi, Frank Peters-Klimm

**Affiliations:** Department of General Practice and Health Services Research, Heidelberg University, Im Neuenheimer Feld 130.3, 69120 Heidelberg, Germany

**Keywords:** COVID-19, Pandemic, Surveillance tool, Care tool, Management, General practitioner, Symptom diary, Outpatient sector

## Abstract

**Background:**

A SARS-CoV-2 infection can lead from asymptomatic through to critical disease in a dynamic and unpredictable course within a few days. The challenge in outpatient monitoring the highly contagious COVID-19 disease during the ongoing pandemic is to filter severe courses followed by admission to hospital with the aim of preventing an overburdening of clinics. However, little is known of the effect of risk factors on the course of the infection of outpatient patients. To support general practices in managing high risk patients, we designed a COVID-19 surveillance and care tool (CovidCare). It includes an initial assessment of yet known risk factors and symptoms and a continuous telephone monitoring of signs and symptoms. This study aims to investigate the effects of different risk factors on the course of the COVID-19 disease, utilisation of different health care services and to gain insights into the utilisation of CovidCare in general practices.

**Methods:**

We will conduct a multi-centered prospective, longitudinal non-controlled observational trial of COVID-19 patients in general practices. Overall, 700 GPs who participate in general-practice centered care by the AOK Baden-Württemberg (large German sickness fund) are eligible and will be invited for study participation, including adult, outpatient COVID-19 patients (or urgent suspicion and ≥ 50 years) with at least one additional known risk factor, who participate in general-practice centered care. The primary outcome is hospitalisation due to COVID-19. Secondary outcomes are diagnosis of pneumonia, utilisation of palliative care, mortality rate, anxiety and identification of predictive risk factors. Quantitative data analysis will focus on valid descriptive figures and mixed regression models. The accompanying process evaluation is based on interviews and questionnaires from general practice staff and patients. The analysis of the process evaluation is descriptive and explorative.

**Discussion:**

The use of the COVID-19 surveillance and care tool is expected to encourage the provision of structured quality of care during the ongoing pandemic. This trial will provide an understanding of the COVID-19-disease and the effect of several risk factors on the course of the disease and health care utilisation. The results can be used for a better management of the COVID-19 pandemic and its consequences.

**Trial registration:**

German Clinical Trials Register DRKS00022054.

**Supplementary Information:**

The online version contains supplementary material available at 10.1186/s12875-021-01515-8.

## Background

Since late December 2019, the newly emerged virus SARS-CoV-2 spreads across the globe, causing the COVID-19 disease. Having started in Wuhan, China, SARS-CoV-2 has spread rapidly, causing catastrophic states for health systems, economies and politics. SARS-CoV-2 causes respiratory symptoms such as coughing, a sore throat, and fever, and can lead to mild, severe and critical disease in a dynamic course over a few days. Several risks associated with a worse course have been identified, such as age (50 years and older), comorbidities (such as diabetes, cardiovascular disease, pulmonary disease), oncological diseases, immunodeficiency, obesity or nicotine consumption. However, also young patients without risks sometimes show a severe to critical course [1, 2]. Patients with severe symptoms are treated in hospitals, often in intensive care units. However, for the majority of patients hospitalisation is not indicated [3, 4] and they remain in their home in a status of officially ordered quarantine. The responsible care provider for these patients remains their general practitioner (GP). In order to prevent healthcare systems to be overwhelmed, patients with mild diseases should be treated by outpatient care providers and more severe courses need to be detected at an early stage and responded to appropriately in a timely manner. However, little is known of the effect of the risk factors identified on the course of the disease. There is a wide range of various models for diagnosing COVID-19, predicting the prognosis of patients with COVID-19 or being admitted to hospital for COVID-19 [5, 6] as well as models to support risk stratification scores [7]. In Germany, the indication of a hospital admission is decided by the outpatient doctor dependent upon age, comorbidities, breathing rate and oxygen saturation [8]. Guidelines on using drugs for the treatment of COVID-19 have also been published and are continuously updated, but are still limited to symptomatic and risk adapted anticoagulation in the outpatient setting [9]. However, it requires a comprehensive approach for the detection and treatment of COVID-19 symptoms. Moreover, in order to keep infection risks in general practices to a minimum, a change of the traditional consultation is necessary. Telemedicine based approaches such as remote monitoring has shown to be effective as in-person care [10, 11]. Therefore, in the presented study data from a COVID-19 surveillance and care tool, that is used in general practices to support outpatient care for patients with increased risk, including structured telephone assessment and monitoring, are analyzed in order to gain a more comprehensive understanding of the COVID-19 disease.

### Objectives

The overall aim of the study is to gain insights into the effects of different risk factors on the course of the COVID-19 disease and the utilisation of different health care services. The accompanying process evaluation aims at gaining insights into the utilisation of the surveillance and care tool in general practices.

## Methods/design

### Trial and study setting

This is a multi-center prospective longitudinal non-controlled observational study of COVID-19 patients exploring the effect of different risk factors on disease progression. The process evaluation follows an exploratory sequential mixed-methods design, qualitative data from interviews and quantitative data from questionnaires and the CovidCare-module. After all qualitative data have been collected, analysed and important aspects have been identified, questionnaires will then be developed based on the qualitative findings to further enrich the results. The interview guides and questionnaires have not been published previously.

### Eligibility criteria {10} and informed consent

All GPs who participate in general-practice centred care (GP-centred care) by the AOK Baden-Württemberg care are eligible for using the CovidCare-module within the CareCockpit software. The eligible practices are informed by the GP-centred care contract partners about the possibility to use CovidCare for the care of confirmed and suspected COVID-19 patients.

Prerequisites are the online registration and participation in an online training including exam questions for the handling of the software. After the successful completion of the exam, general practices can download the software.

All general practices who have successfully completed the exam and who download the software are automatically provided with an invitation for participation in the CovidCare study as well as an information leaflet and a participation form.

General practices can participate in the CovidCare study by sending the signed participation form to the study central office (Department of General Practice and Health Services Research, University Hospital Heidelberg).

Additional time expenditure for GP-practices for participation in the study concern information of eligible patients, obtaining the informed consent as well as the weekly data transfer. Time expenditure for this study depends on the number of patients a general practice can include in the study.

The study population comprises of COVID-19 patients treated in general practices who meet the inclusion criteria. The study population for the process evaluation consists of GPs, VERAHs (Care Assistant in General Practice (*Ver*sorgungs*a*ssistentin in der *H*ausarztpraxis)) and patients participating in the CovidCare study.

The following groups of patients are eligible for participating in the study:

1. COVID-19 patients aged 18 or older (as per definition patients with symptoms of an infection of any severity and positive test for SARS-CoV-2), who are in domestic isolation* and who have at least one risk factor**

OR

2. Patients aged 50 and older with a clinically urgent suspicion of COVID-19 (such as acute respiratory symptoms of any severity), without verification of SARS-CoV-2 and who have at least one risk factor**/***

*Domestic isolation: includes persons for whom the authorities highly recommend further social distancing beyond the restrictions as currently ordered.

*** This criterium might by subject to potential changes and might be further adapted according to (a) the current epidemiological situation, (b) the requirements of the Robert-Koch-Institute (RKI), (c) the test strategy as well as (d) the test capacity.

**Risk factors following the definition by the RKI, with own additions:

- Diabetes mellitus (Types I and II)
- Cardiovascular disease (arterial hypertension, coronary heart disease, heart failure, other such as atrial fibrillation, peripheral artery disease, condition after apoplexy)
- Pulmonary disease (COPD, bronchial asthma, other)
- chronical nephrological disease
- chronical hepatic disease such as liver cirrhosis
- immunodeficiency due to underlying disease (malignant primary hematological disease (e.g. leukemia/ lymphoma), leukocytopenia, condition after organ or stem cell transplantation or potentially immunosuppressive medication intake such as glucocorticoids, immunosuppressive drugs, immunomodulators, cytostatics
- active oncological disease with immunodeficiency (excluding for example localized skin cancer such as squamous cell carcinoma or basal cell carcinoma)
- obesity (body mass index > 35)
- active smoking
- Down syndrome

All patients have to be 18 years and older, insured with the AOK Baden-Württemberg, participate in GP-based care, and have written and spoken German language skills.

If patients are unable to give their informed consent into study participation, a legal representative of the patient has to give their informed consent for study participation of the patient.

Participants for the process evaluation have to participate in the CovidCare study as either patient or health care provider, are 18 years and older, have written and spoken German language skills and have to be able to give their informed consent.

No further exclusion criteria have been defined.

## Intervention

### Explanation for the choice of comparators

Not applicable as we did not choose any comparators.

### Intervention description

Measures within the CovidCare-module include patient intake, an initial assessment and several telephone monitoring calls. In the initial assessment, information on risks (age, obesity, nicotine consumption, diabetes, cardiovascular disease, pulmonary disease, chronical hepatic disease, chronical nephrological disease, immunodeficiency, treatment of oncological disease with immunodeficiency, vaccination status), gender, SARS-CoV-2 status, living condition and SARS-CoV-2 status of contact persons, symptom history, legal information (health authorisation, patient decree) is collected and inclusion in the telephone monitoring is defined. The GP individually sets the presumable date of onset of COVID-19 and the extent and frequency of the telephone monitoring. GP-ascertained begin of COVID-19 equals to day 1. GPs are responsible to consider the appropriate monitoring intervals referring to the actual clinical knowledge base regarding the disease course with milestones for deterioration and recovery.

Patients are provided with a monitoring protocol that can be individualized, e.g. for daily log of symptoms and vital signs. The telephone monitoring includes a monitoring of symptoms and signs of infection and the cardio-respiratory system. Individual timelines and figures are created and provide an overview on first symptoms (presumable disease onset), positive test result, disease progression. This aids in assessing the state of health and early detection of a deterioration. A total number of at least three to four contacts is recommended, including the intake, the initial assessment and a closing contact. Any further contacts should be conducted in the critical phase as defined by the knowledge base provided by the RKI [12, 13], the frequency is to be increased with a deterioration. The closing contact includes a short closing documentation not later than three weeks after disease onset. In this closing contact, information on the utilisation of different health care services (e.g. hospitalisation, ventilation) are retrieved and documented.

No further telephone monitorings are conducted when (a) the patient is cured (defined as alive and without symptoms of COVID-19 for 48 h and longer), (b) the patient is hospitalised, (c) the patient has passed away, (d) none of the above has happened not later than three weeks after first symptoms showed.

For patients who are not able to give their informed consent into study participation and where a legal representative gives their informed consent or for patients who are residing in a retirement home or a long-term care institution, the responsible nurse or the responsible legal representative or informal caregivers can document symptoms and vital signs in the monitoring protocol. If none of the above is possible, information can be extracted from existing nursing documentation. For the same groups of patients, the telephone monitoring can be conducted with the responsible nurse or the responsible legal representative or an informal caregiver, if necessary.

The CareCockpit software provides a schedule that allows for systematic follow-ups according to the defined telephone monitoring intervals.

### Criteria for discontinuing or modifying allocated interventions

Not applicable as this is a non-intervention study.

### Strategies to improve adherence to interventions

Not applicable as this is a non-intervention study.

### Relevant concomitant care permitted or prohibited during the trial

Not applicable as this is study does not investigate the use of drugs.

### Provisions for post-trial care

Not applicable as no risks are anticipated for patients and general practices by participating in this study.

### Outcomes

The primary outcome is hospitalisation due to COVID-19. The following indicators have been identified as secondary outcomes:

1. Diagnosis of pneumonia in confirmed and suspected COVID-19 patients [14].
2. Utilisation of palliative care by confirmed and suspected COVID-19 patients during quarantine or stay at hospital.
3. Mortality rate of confirmed and suspected COVID-19 patients (death at home, death in hospital, death in long-term care institution).
4. Anxiety in confirmed and suspected COVID-19 patients using the Generalized Anxiety Disorder Index (GAD-2) [15].
5. Identification of predictive risk factors for the outcomes such as hospitalisation, intensive care and mortality.

For the process evaluation we chose an exploratory sequential design. The process evaluation consists of a qualitative strand and a quantitative strand.

Overall, for the process evaluation we use the RE-AIM framework according to Glasgow and colleagues [16] to assess reach, efficacy, adoption, implementation and maintenance of the CovidCare surveillance and care tool. Table 1 provides an overview of the outcomes, indicators and methods/instruments of the process evaluation according to the RE-AIM framework.

**Table 1:** Concept of the process evaluation

Outcomes	Indicators (examples)	Methods/Instruments
1) Reach The extent to which patients are reached by the implementation of CovidCare as well as the number of patients who are willing or refused to participate in CovidCare.	• How have patients been approached • Number of patients approached • Number of patients willing to participate in CovidCare • Patients: Reasons for refusal and drop-out, exclusion rate • Number of contacts per patient (i.e. telephone monitoring, particularly in the critical phase as defined by the RKI as a proxy whether GPs treat their patients according to guidelines) • Characteristics of participating patients	S, I, DB
2) Efficacy The efficacy of the CovidCare-module includes positive and negative impacts/effects for patients and HCP as well as the patients’ quality of life and the patients and the HCP’s satisfaction with the intervention.	• Positive and negative effects on patients’ quality of life, perception of (quality of) care, disease coping • Effects on health care personnel in managing Covid-19 patients • Satisfaction of HCP and patients with CovidCare • Comparison with patients with Covid-19/suspected cases but WITHOUT additional risk factors or aged >50	S, I, DB
3) Adoption Adoption refers to the number of HCP and practices who agree to use the CovidCare-module and aims to understand the reasons for (not) participating in the intervention.	• Reasons for (not) participating in CovidCare; comparing characteristics of participants and non-participants (HCP, practices and patients) • Characteristics of participating HCP and practices • Barriers and facilitators	I, S, DB
4) Implementation Implementation refers to the extent to which CovidCare is used delivered as intended.	• Problems, time, and cost of the intervention • Adaptions that were necessary to implement/use CovidCare in daily practice	I, S, DB
5) Maintenance The extent to which the CovidCare-module became part of the daily practice routines of the individual HCP and the GP’s practice.	• Factors that facilitate the uptake of the intervention/ implementation into routine care • Suggestions for improving the intervention in order to enable CovidCare to become part of organizational practice	I

*DB* database, *HCP* health care personnel, *I* interviews, *S* survey

### Participant timeline

Development of study material, such as assessments, and software programming has started in March 2020. The CovidCare-module is available for eligible general practices since April 30th 2020. Patient recruitment for this study in the general practices can start as soon as the software has been installed and activated. Data collection ends on December 31st 2021. The process evaluation will start in March 2021. All patients whose COVID-19 disease have been cured will be invited to participate in an interview and in the questionnaire survey after the final consultation. Patients willing to participate in the process evaluation will be contacted by the study team via telephone to get more information about the study, answer open questions and schedule an interview and send the questionnaire to the patient. Interviews will take place in a timely manner after the patient provided his/her written informed consent. Evaluation, data analysis and publication are planned to be conducted continuously. If the COVID-19 pandemic persists or becomes active again the study may be prolonged after positive verification of an amendment to the ethics committee. In this case the practices have to reactivate the software with a new key after re-registration.

### Sample size

The sample size of this observational study cannot be determined as it depends on the incidence of COVID-19 in the population and the further spread of SARS-CoV-2 is uncertain.

Currently, approx. 700 general practices use the CareCockpit software in Baden-Wurttemberg. It is expected that more practices will use the CareCockpit in the coming months. The maximum is limited by the number of practices who provide a trained VERAH. This number is presumed to be 2.000 practices. We expect participation in the CovidCare Study to be above 10 % of the existing 700 practices.

The sample for the qualitative study within the process evaluation is planned to reach saturation of data; a total of *n* = 20 (15–25) GPs and/ or VERAHs and *n* = 20 (15–25) patients is expected to be sufficient. The sample for the quantitative questionnaire survey comprises of all participating health care personnel and patients.

### Sequence generation

Not applicable as this is not a randomized controlled trial.

### Recruitment {15} and Implementation

General practices are study sites and will check the inclusion criteria for all confirmed and suspected COVID-19 patients who they have included in the CovidCare-module during a phone call. The general practice then provides the patient/ legal representative with the study information and informed consent form via post or family members/ other persons supporting the patient in their quarantine.

 The patient/legal representative signs the informed consent form after being informed by the general practice via telephone. The signed informed consent form is transferred back via post or family members/ other persons supporting the patient in their quarantine.

If patients are in a temporary state of not being able to give their informed consent, for example due to invasive ventilation, a legal representative will be able to sign the informed consent and informed consent by the patient will be obtained once this temporary state is over.

 The general practice documents that they have sent the informed consent documents to the patients as well as that they have received the signed informed consent form in the CovidCare-module in the CareCockpit software. No data is transferred from the CovidCare-module if informed consent was not received by the practice and if the corresponding box in the software is not ticked.

The only additional time expenditure for patients concerns the informed consent.

For participation in the process evaluation, all participating GPs and VERAHs in the CovidCare study will be invited by an official letter from the study central office and will be asked to participate in an interview or the questionnaire survey, respectively.

For participation in an interview, eligible GPs and VERAHs will receive a response coupon with which they can declare their interest in participating in an interview. They will e-mail, fax or mail (using an enclosed post-paid envelope) the response coupon to the study central office. An employee of the study central office will call all interested participants in order to give further information on study participation, answer any open questions and arrange interview appointments. They will then be provided with a study information and the written consent form. The participant sends the signed informed consent sheets to the study central office using an enclosed post-paid envelope. The interviewer signs the form as well and sends back a copy to the participant.

For participation in the questionnaire survey, eligible GPs and VERAHs will be provided with a study information, informed consent forms and paper-based questionnaires. Interested persons will send the filled-in questionnaire and signed consent form in an enclosed post-paid envelope to the study central office.

As for patients, all participating patients will be informed about participation in an interview and in the questionnaire survey by the VERAH after the final consultation. The VERAH will send an expression of interest form and a post-paid envelope to the patients interested in participating in an interview and/or the questionnaire survey. The patient sends the expression of interest forms to the study central office using the enclosed post-paid envelope and will then be contacted by the study team via telephone to get more information about the study, answer open questions and schedule an interview. The study team will send the informed consent forms for the interview and the questionnaire respectively (each in duplicate), the study information and one post-paid envelope to the patient who will sign and return all informed consent forms to the study central office for study participation. A member of the study team will then sign all informed consent forms and return one copy (of the informed consent) to the patient and send him/her the questionnaire via post.

### Who will be blinded {17a} Procedure for unblinding if needed

Not applicable, as this is not a blinded trial.

### Data collection and management

#### Plans for assessment and collection of outcomes

Data sources for the evaluation are data collected within the CovidCare-module within the CareCockpit software in general practices in Baden-Wurttemberg. The CareCockpit software, developed and licensed by the Department of General Practice and Health Services Research at the University Hospital Heidelberg, is designed for case management in general practices and is currently used in approx. 700 general practices in Baden-Wurttemberg. The CovidCare-module therefore is an extension of the electronic patient record in general practices and supports GPs to take care of confirmed and suspected COVID-19 patients in a structured and standardized way. The CovidCare-module has been recently developed and is implemented in general practices within GP-centred by the AOK Baden-Württemberg.

The following data will be recorded from general practices: ID, practice identification number, date and time of the dataset created.

In an initial assessment and telephone monitoring, potential risk (risks) factors are recorded and symptom progression is documented.

Consequently, the following data will be recorded from patients:

ID, gender, date of the informed consent, treatment ended (yes/no), date of end of treatment as well as reasons, request to delete data (yes/no), date and time of the dataset created; data on their individual assessments (examples: ID, date of assessment, ID of VERAH who conducts the assessment, age, diabetes, cardiovascular data, data on pulmonary diseases, liver diseases, immune system, medication, cancer diseases, other diseases, hospitalisation, obesity, smoking, pneumococci, influenza, medication regimen) and monitoring, when available (examples: ID, date and time and place of the dataset created, ID of VERAH who conducts the monitoring, anxiety, medication, patients’ condition as well as symptoms and course of the disease, cured, deceased).

Data sources for the process evaluation are questionnaires and qualitative telephone interviews with GPs, VERAHs and patients using self-developed semi-structured interview guides (see Additional files 1 and 2) and paper-based questionnaires according to the RE-AIM framework (see Table 1) as well as data from the CovidCare-module. Self-developed questionnaires will be presented to the ethics committee in an amendment.

Interviews will be audio-recorded and then transcribed by research assistants of the Department of General Practice and Health Services Research, University Hospital Heidelberg. Interviews will be complemented by sociodemographic characteristics of the participants and information on the associated general practices. Questionnaires are digitalized for the purpose of data analysis.

#### Plans to promote participant retention and complete follow-up

General practices are study sites and are responsible to provide continuous telephone-monitoring. The following data will be collected from all study participants: ID, gender, date of the informed consent, treatment ended (yes/no), date of end of treatment as well as reasons, request to delete data (yes/no), date and time of the dataset created; as well as data on their individual assessments and monitoring, when available. The following data will be collected from all study sites/general practices: ID, practice identification number (Betriebsstättennummer, BSNR), date and time of the dataset created.

#### Data management, Confidentiality

The evaluating institution (Department of General Practice and Health Services Research, University Hospital of Heidelberg) receives pseudonymized data only and cannot assign data to individuals.

Data collected in the CovidCare-module are secured in the general practices.

Pseudonymized data from patients who have given their informed consent is transferred to the study central office weekly. Data include all pseudonymized data collected in the initial assessment and the telephone monitoring as described in the intervention description.

Patients in each general practice are given a sequential number, which is linked to the corresponding general practice in the data. For the purpose of the identification of general practices, they export their unique license key as well as their practice identification number. In the study central office, a list linking the license key and the BSNR to a practice name and address data is stored separately and secured.

Data will be transferred via a secure unidirectional way (Zentraler Daten-Austausch-Container/ Kryptographie- und Transfer-Modul, ZeDaC-KTM). The ZeDaC-KTM interface allows for flexible, universal, and secure data exchange between health care organisations and their partners (here: Dept. of General Practice and Health Services Research). Hybrid encryption is used, which combines the benefits of asymmetric (public-key cryptosystem RSA (4096 bit)) and symmetric encryption (Advanced Encryption Standard (AES) 256 bit). The key length lies above the recommendations of the Federal Office for Information Security. This means that data that is temporarily stored on the ZeDaC-KTM server for the purpose of data transfer is encrypted and not readable. Only when data is collected from the server by the study central office, the encryption is reversed.

Digital data from questionnaires, interviews, and the CovidCare software module is stored on database-servers at the Department of General Practice and Health Services Research, University Hospital Heidelberg and are only accessible by the study team. Servers are in a separate room that can only be accessed by authorized personnel of the IT-team. Servers are not connected to the internet and cannot be accessed externally. All system users must have personalized access to the network of the University Hospital Heidelberg. Additionally, users must have a personalized user account in order to access the server. Data on the server is secured on the data storage of the data center of the University Hospital Heidelberg on a daily basis.

Data is secured for 10 years after the completion of the study or until a participant withdraws his/her consent to participate and demands to have data already collected destroyed, and will then be destroyed irretrievably.

Anonymized data only will be used for publications.

The existing data protection concept of the CareCockpit, proven by all partners will be used.

The principle investigator is responsible for data processing.

#### Plans for collection, laboratory evaluation and storage of biological specimens for genetic or molecular analysis in this trial/future use

Not applicable, as this is not an ancillary study.

### Statistical methods

#### Statistical methods for primary and secondary outcomes

Data analysis is explorative. Date of the full study population (general practices and patients) will be included in data analysis.

Descriptive statistics of all collected data will be performed. Continuous variables will be described using means, standard deviations, median, minimum, maximum, and first and third quartile. Categorical variables will be reported using absolute and relative frequencies.

The potential effect of sociodemographic characteristics (age, gender, living situation), risk factors (as described above) on the severity of the disease, utilisation of different care services (hospitalisation, intensive care unit, invasive ventilation, palliative care) will be estimated by models taking the clustered structure into account. Therefore, e.g. SAS PROC MIXED, PROC GENMOD or PROC GLIMMIX can be accordingly parametrized to determine fixed and random effects. In order to determine potential differences concerning mortality between particular strata there are also a couple of methods available to take a clustered structure into account. One option is a likelihood-based random effects (frailty) model. The baseline hazard could be either a priori determined (e.g., Weibull) or approximated by piecewise constant counterpart. The estimation could be carried out by adaptive Gaussian quadrature method which is implemented in SAS PROC NLMIXED.

Incubation, begin of the disease, and progression of the disease will be analysed descriptively.

Data analysis for the process evaluation is descriptive and explorative. Qualitative data from interviews will be transcribed according to established standards and will be analyzed with regard to the research questions with qualitative content analysis [17] or framework analysis using the software MAXQDA. Quantitative survey data and the indicators for the intervention fidelity will be analyzed descriptively.

#### Interim analyses

Interim analyses are not planned. No risks are anticipated for patients and general practices by participation in this study. If -against expectation- circumstances should arise which suggest to terminate the study, the primary investigator is the decisive authority for a termination decision.

#### Methods for additional analyses (e.g. subgroup analyses)

Descriptive subgroup analyses are planned, especially in terms of patients’ age, gender, and risk factors. Adjustment of inference will be performed by multivariable regression analyses as described above.

#### Methods in analysis to handle protocol non-adherence and any statistical methods to handle missing data

The protocol non-adherence is limited to non-response, especially loss-to-follow-up. If necessary, a non-responder analysis will be performed. The data-sets themselves will obtain no missings due to mandatory fields. Therefore no imputation techniques will be needed.

#### Plans to give access to the full protocol, participant level-data and statistical code

It is planned to publish the study results in high ranked international journals. According to the journal guidelines, full protocol, participant-level dataset and/or statistical code may be granted public access, depending on the journal’s guidelines.

### Oversight and monitoring

#### Composition of the coordinating centre and trial steering committee

The coordinating centre consists of a team of researchers, medical doctors, and study nurses. FPK is Chief Investigator, he conceived the study together with all other authors. GL will be responsible for quantitative data analysis and MH will conduct the qualitative data collection as well as data analysis in coordination with SS. AB will be responsible for the administration of the general practices/study sites in coordination with MH. SZ and FPK will be expert consultants regarding all aspects of the study.

#### Composition of the data monitoring committee, its role and reporting structure

Not applicable as this is not a clinical trial and no risks are anticipated in association with the CovidCare-Module.

#### Adverse event reporting and harms

No risks are anticipated for patients and general practices by participating in this study. However, we are in constant contact with all study sites to ensure that the CovidCare-Module can be used as intended and to early identify and solve occurring problems. All study sites are encouraged to report any difficulty to the study team. Moreover, we will explicitly explore problems and adverse events as part of the process evaluation, namely the interviews with the GPs, VERAHs and patients and report them in corresponding publications. Patients and general practices participating in this study can withdraw their consent at any time without indication of reasons. If the VERAH stops working at a general practice and there is no longer any VERAH employed, the GP-practice cannot participate in the CovidCare study any longer. New insights and findings of other studies that make the conduct of this study dispensable will lead to study termination.

#### Frequency and plans for auditing trial conduct

See item 22. The sponsor does not have any influence in conducting the study.

#### Plans for communicating important protocol amendments to relevant parties (e.g. trial participants, ethical committees)

 Any modifications to the protocol which may influence the impact of the CovidCare-Modul, the study objectives, the study sample or other aspects of the study will be submitted to and approved by the ethics committee of the Medical Faculty Heidelberg as well as to the ethics committee of the State Medical Council of Baden-Württemberg. Any resulting changes will be agreed upon by the research team and will be made transparent to the public in a memorandum.

#### Dissemination plans

The study results will be presented in corresponding publications preferably within open access journals in order to make them available to a wide range of researchers, healthcare professionals, and health policy makers. Moreover, the results will be provided to the participating study sites and the participants.

## Discussion

The COVID-19 surveillance and care tool allows to monitor the course of COVID-19 and to adjust and tailor the treatment promptly as soon as symptoms worsen. This encourages the GP and VERAH in providing structured and high-quality care. Regarding the study participation in particular, no personal effect by participating in this study is expected.

Overall, this study is expected to gain understanding of the COVID-19-disease and the effect of several risk factors on the course of the disease and health care utilisation. The results can be used for a better management of the COVID-19 pandemic and its consequences.

### Trial status

Protocol version number: 1.4, date: 19th May 2021.

Date the recruitment began: 30th April 2020.

The approximate date when recruitment will be completed: 31st December 2021.

## Supplementary Information

**Additional file 1.**

**Additional file 2.**

## Data Availability

The full datasets generated and/or analysed during the current study are not publicly available due privacy reasons of the individual participant. However, data generated and/or analysed during this study will be included in articles resulting from this study. For example, we will include quotations from study participants’ interviews in articles on the process evaluation.

## Abbreviations

AOK: Allgemeine Ortskrankenkasse, large German sickness fund
GP: General practitioner
RKI: Robert-Koch-Institut
VERAH: Care Assistant in General Practice (*Ver*sorgungs*a*ssistentin in der *H*ausarztpraxis)
