# Supplementary material for: Surveillance and care for confirmed and suspected patients with COVID-19 in general practice (CovidCare): study protocol for an observational trial
Source: BMC Fam Pract. 2021 Sep 2;22:173. doi: 10.1186/s12875-021-01515-8 (PMC8412868; doi:10.1186/s12875-021-01515-8)
Supplement: Supplementary file 1 — Additional file 1. [file 12875_2021_1515_MOESM1_ESM.pdf]

Process evaluation:

Surveillance and care for confirmed and suspected patients with COVID-19 in general practice (CovidCare)

Interview guide for patients

(finalised version as of 31st January 2021)

1. Reach

- How would you describe your willingness to be treated in the CovidCare-module and participate in the CovidCare study?
- How were you made aware of the study?
- How many Assessments and Monitorings have been conducted?

2. Adoption

- What motivated you to use the CovidCare-module and participate in the CovidCare study?
- What was your first impression of the CovidCare-module?
    - Which expectations and concerns did you have?
    - What has (not) been fulfilled?
- What advantages and disadvantages do you anticipate in using the CovidCare-module?

3. Efficacy

- How satisfied were you with the treatment of the CovidCare-module?
    - e.g. duration of the Assessments/Monitorings
- Which aspects of the CovidCare-module have you used (e.g. symptom diary)?
    - Which aspects did you find (not) helpful and why?
- To what extent has the treatment within the CovidCare-module influenced you? Do you consider this as helpful, why?
    - To what extent has the treatment within the CovidCare-module influenced the relationship to your general praticioner?
- To what extent has the CovidCare-module contributed to your management of your Covid-19 disease?

4. Implementation

- How did the Assessment or Monitoring proceed?
    - Who conducted the Assessment/Monitoring/final consultation?
    - What were you advised to do?
    - Which of these aspects have you implemented?
- How were you cared for by your general practicioner before using the CovidCare-module?
- To what extent have you perceived problems regarding the treatment within the CovidCare-module?

5. Maintenance

- Would you recommend care via the CovidCare-module to others?
    - Why (not)?
- What would you suggest to facilitate the usage of the CovidCare-module?
- Which importance do you attribute to app-based applications?

6. Interview termination

- Are there any remaining aspects important to you that we have not addresses so far?
- Do you have any further questions?
