# Supplementary material for: Surveillance and care for confirmed and suspected patients with COVID-19 in general practice (CovidCare): study protocol for an observational trial
Source: BMC Fam Pract. 2021 Sep 2;22:173. doi: 10.1186/s12875-021-01515-8 (PMC8412868; doi:10.1186/s12875-021-01515-8)
Supplement: Supplementary file 2 — Additional file 2. [file 12875_2021_1515_MOESM2_ESM.pdf]

Process evaluation:

Surveillance and care for confirmed and suspected patients with COVID-19 in general practice (CovidCare)

Interview guide for general practitioners and VERAHs

(finalised version as of 31st January 2021)

1. Reach

- What motivated you to use the CovidCare-module and participate in the CovidCare study?
- Only for general practitioners: How did you decide whether or not a patient will be monitored within the CovidCare-module/will be invited to participate in the CovidCare study?
    - How were patients made aware of the study?
    - Were patients deliberately not made aware of the study?
- How would you describe the willingness of patients to be treated in the CovidCare-module and participate in the CovidCare study?
- How would you describe the patients who have been treated within the CovidCare-module?

2. Efficacy

- How satisfied were you with the use of the CovidCare-module?
- Which aspects of the CovidCare-module have you used?
- Which aspects did you find (not) helpful and why?
- To what extent has the use of the CovidCare-module influenced patient care? Do you consider this as helpful, why?
    - Which effects did you recognize for patients? Do you consider this as helpful, why?
- To what extent has the use of the CovidCare-module influences your work (positively and negaltively)?

3. Adoption

- Why did you decide to implement the CovidCare-module?
    - Which expectations and concerns did you have?

    - What has (not) been fulfilled?

- What advantages and disadvantages do you anticipate in using the CovidCare-module?
- Why did some general practitioners and/or VERAHs in your practice use the CovidCare-module and others not?

4. Implementation

- How do you use the CovidCare-module? Can you describe the process with an example of a patient?
    - Who conducted the assessment, monitoring etc.?
- To what extent have you made adjustments? (e.g. regarding the number of assessments, monitorings and the questions)
- How did you treat Covid-19-patients before using the CovidCare-module?

5. Maintenance

- Will you continue to use the CovidCare-module in your practice? Why (not)?
- How do you assess the chances of a widespread implementation of the CovidCare-module?
    - Which facilitators and barriers do you anticipate?
- What would you suggest to facilitate the usage of the CovidCare-module?
- Which importance do you attribute to app-based applications?

6. Interview termination

- Are there any remaining aspects important to you that we have not addresses so far?
- Do you have any further questions?
